# Supplementary material for: An Approach for Detecting Mangrove Areas and Mapping Species Using Multispectral Drone Imagery and Deep Learning
Source: Sensors (Basel). 2025 Apr 17;25(8):2540. doi: 10.3390/s25082540 (PMC12031454; doi:10.3390/s25082540)
Supplement: Supplementary file 1 [file sensors-25-02540-s001.zip › sensors-3495532-supplementary.pdf]

Supplementary material for

An Approach for Detecting Mangrove Areas and Mapping Species Using Multispectral Drone Imagery and Deep Learning

Xingyu Chen [1, 2], Xiuyu Zhang [1, 2, *], Changwei Zhuang [1, 2], Xuejiao Dai [1, 2], Lingling Kong [1, 2], Zixia Xie [1, 2], and Xibang Hu [1, 2]

1 Institute of Ecological Civilization and Green Development, Guangdong Provincial Academy of Environmental Science, Guangzhou 510045, China;

2 Ecological Environment Remote Sensing Research Center, Guangdong Provincial Academy of Environmental Science, Guangzhou 510045, China

* Correspondence: zhangxiuyu@scies.org

Section A: Comparison of the effects of each attention module in mangrove extraction.

We designed a comparison experiment to compare the difference between using only spectral attention, using only spatial attention, and using both attention modules. We found that the effect of using a single attention module was slightly lower than that of using two attention modules at the same time, such as 0.9875 miou when using only spectral attention, 0.9863 miou when using only spatial attention, and a higher miou of 0.9882 when using both spectral and spatial attention modules.

Table S1. Comparison of the effects of each attention module in mangrove extraction.

**Model Structure**	**Accuracy**	**F1_Score**	**mIoU**	**Precision**	**Recall**
only spectral attention	99.42%	99.25%	98.75%	98.79%	99.89%
only spatial attention	99.33%	99.19%	98.63%	98.68%	99.89%
using both attention modules	99.46%	99.32%	98.82%	99.49%	99.25%

Section B: How attention mechanisms enhance mangrove boundary detection.

Through the spatial attention mechanism, the model can dynamically assign weights to different spatial positions, and prioritize the regions with significant edge features. For example, in complex background, this mechanism can effectively suppress irrelevant region interference and make the boundary response clearer. However, the attention mechanism also has some shortcomings. When the spatial-channel attention mechanism is used jointly, the double computation of feature graph dimensions will significantly increase the inference time and is unfriendly to real-time edge detection.

Section C: The changes of miou and loss among several models.

We compared the changes of miou and loss among several models during the training process. There is no difference in the rate of loss reduction among these models. MangroveNet exhibited the lowest Loss value, while the mIoU index demonstrated the highest rate of increase and reached the highest value in the validation dataset after 20 training rounds, indicating that its generalization ability is stronger than other models.

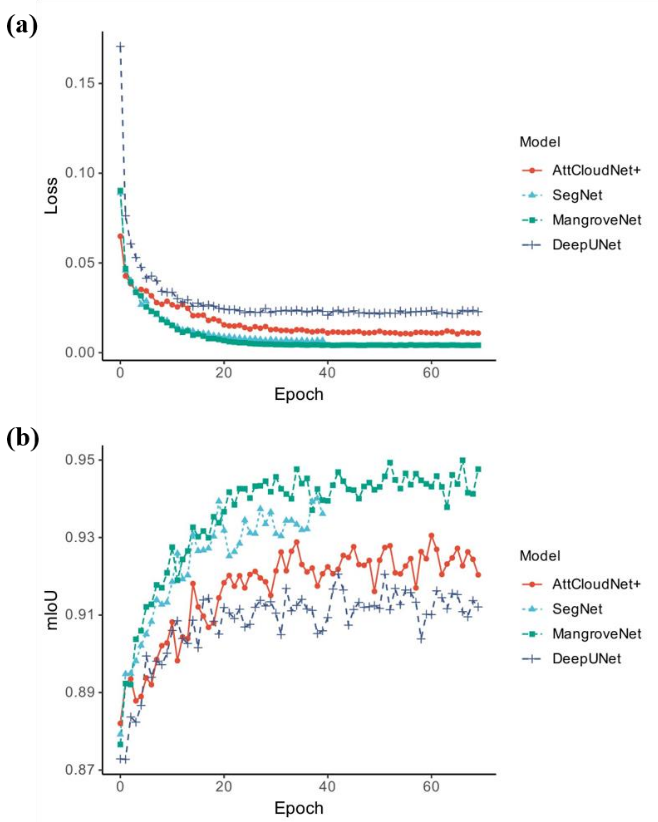

Figure S1. The change of Loss and miou in the validation dataset of each model with the number of trainings.

Section D: Generalization experiment of mangroves extraction in Southeast Asia.

We selected mangrove sample area (13.66°N, 124.35°E) in a coastal region of Southeast Asia as supplementary research. We obtained drone RGB images in this area on the OpenAerialMap website (https://map.openaerialmap.org/) for pre-processing and prediction, so as to test the generalization performance of the model. The results show that our MangroveNet also has a good extraction effect on mangroves in the coastal area of Southeast Asia, but there are also some problems, such as shallow water and other disturbance features are not completely eliminated, which will help us to continue to improve our model in the future to enhance the extraction ability of mangroves in other regions of the world.

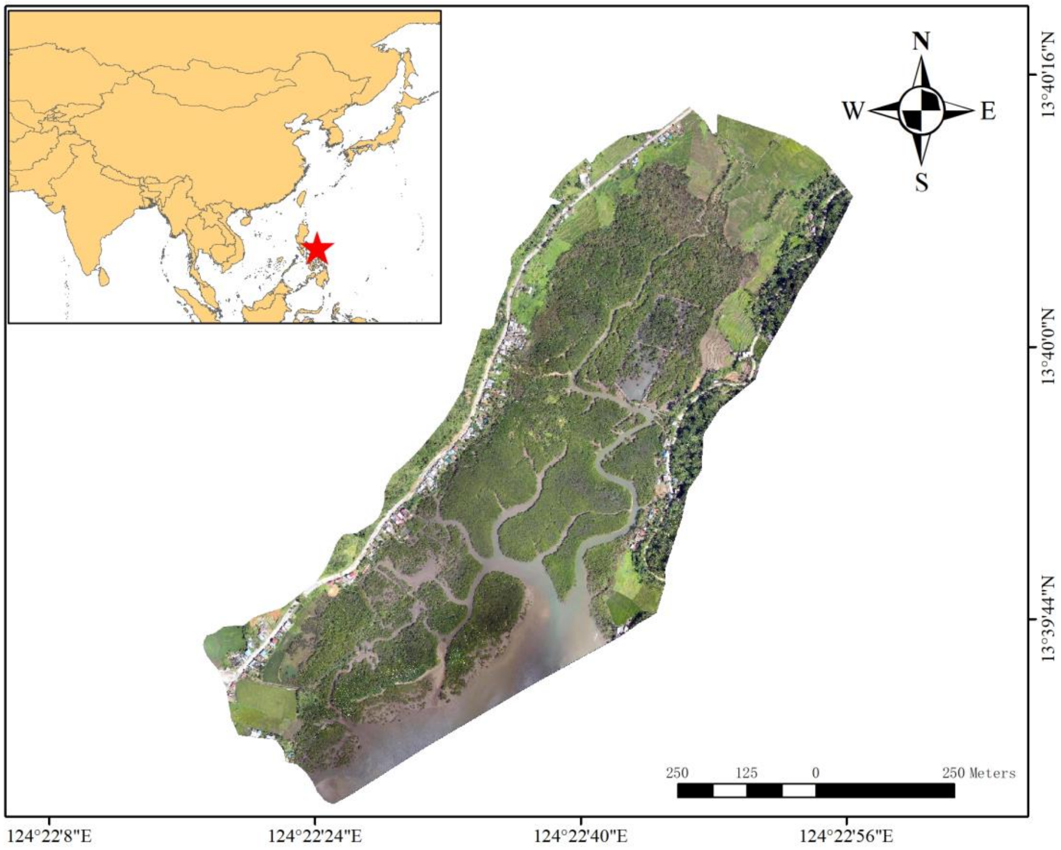

Figure S2. Location diagram of the coastal mangrove area in Southeast Asia.

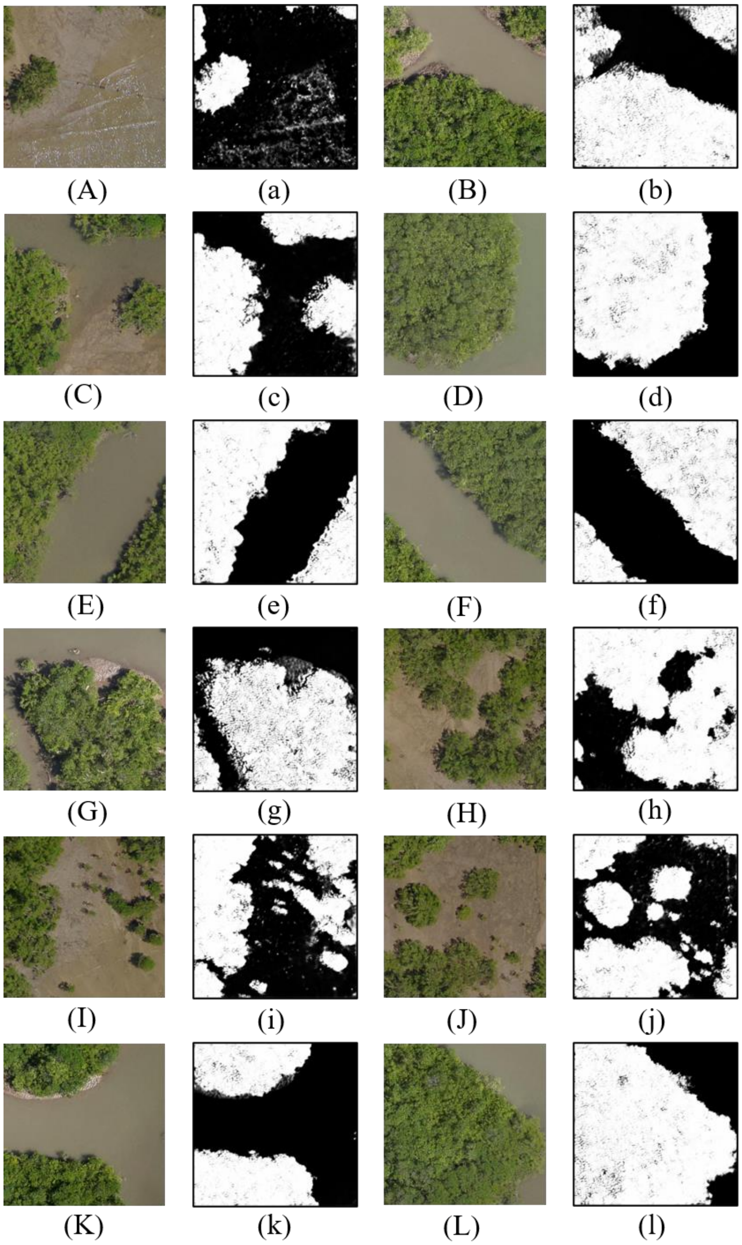

Figure S3. Drone images and model recognition results of mangrove sample area in Southeast Asia. Uppercase letters (A-L) represent the drone images of mangrove areas to be predicted, and lowercase letters (a-l) represent the prediction results.

Section E: Performance on historical lower-resolution satellite imagery.

We try to process low-resolution satellite remote sensing data to extract mangrove areas from satellite images. We selected mangrove satellite images of Jinniu Island in 2012 and 2022 respectively, and after preprocessing them, used the weight parameter file trained by the model to predict them. Finally, the spatial distribution and area size of mangroves in this area were calculated. In 2012, the mangrove forest distribution in the area was 50.326 km^2, and in 2022, the mangrove forest in the area was 47.748

km². The reason for the change may be the degradation of mangroves caused by human development activities such as coastal aquaculture and farming activities.

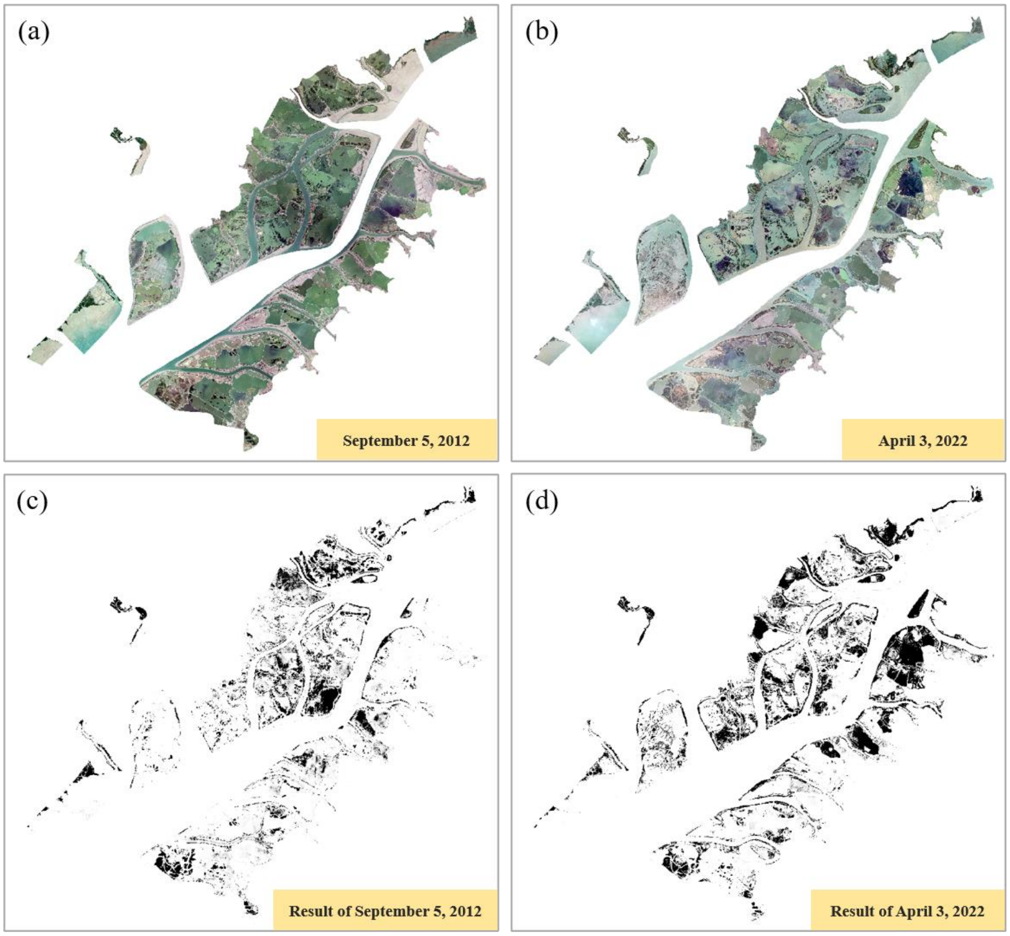

Figure S4. Mangrove identification results from satellite images in 2012 and 2022. (a) and (b) represent the historical satellite images. (c) and (d) are corresponding results of model prediction.
